# Exercise-induced rhabdomyolysis following a swimming session complicated by acute kidney injury: a case report

**DOI:** 10.1097/MS9.0000000000005416

**Published:** 2026-07-28

**Authors:** Mohammad Maraqah, Ameer Shawar, Ammar Shaheen, Sara Abu Aram, Rand Al Atrash

**Affiliations:** a Department of Medicine, Hebron University, Hebron, Palestine; b Department of Internal Medicine, Al-Hussein Governmental Hospital, Bayt Jala, Palestine

**Keywords:** acute kidney injury, exertional rhabdomyolysis, pulmonary edema, swimming

## Abstract

**Introduction:**

Exercise-induced rhabdomyolysis (EIR) is an uncommon but potentially serious condition that can occur after intense or even moderate physical exertion. While EIR is typically linked to strenuous activity or heat exposure, swimming is a rarely reported trigger.

**Case presentation:**

A 34-year-old healthy man developed generalized myalgia, dark urine, and vomiting 1 day after a 2-hour swimming session in hot weather. Laboratory tests revealed markedly elevated creatine phosphokinase (250 000 U/L) and acute kidney injury (peak creatinine 14 mg/dL). He received aggressive intravenous hydration, targeting a urine output of 2 mL/kg/hr, with close monitoring. During hospitalization, pulmonary edema due to fluid overload developed, but it resolved with diuretics and oxygen therapy. The patient achieved complete renal recovery without dialysis.

**Clinical discussion:**

EIR can occur even in healthy individuals after moderate exertion. Marked creatine kinase elevation and acute kidney injury are common findings. While early hydration remains the cornerstone of management, excessive fluids may precipitate pulmonary edema, emphasizing careful titration and monitoring.

**Conclusion:**

Early, aggressive but goal-directed fluid therapy is vital. Monitoring and promptly treating fluid overload are essential, and even severe biochemical derangements may be managed conservatively when dialysis indications are absent, with multidisciplinary critical care and nephrology input recommended.

## Introduction

Exertional rhabdomyolysis (ER) is a well-recognized but uncommon syndrome that typically follows extreme or unaccustomed exercise. In one large U.S. series, the incidence of ER was only about 0.66 per 100 000 persons^[^1^]^. Classic triggers of ER are strenuous muscular activity, particularly in untrained individuals, heavy weightlifting, and exercising in extreme heat^[^2^]^. By contrast, swimming has only rarely been implicated as a trigger. Indeed, to our knowledge, only a handful of cases have been reported in the literature^[^3,4^]^, likely because it involves predominantly concentric rather than eccentric muscle contractions and is performed in a weight-reduced, cooler environment, all factors that reduce the risk of muscle injury^[^4^]^.

HIGHLIGHTS

- Exercise-induced rhabdomyolysis can occur even after moderate physical activity, such as swimming, in healthy individuals without prior risk factors.
- Early recognition and prompt initiation of intravenous fluid therapy are essential to prevent irreversible kidney injury.
- Despite appropriate fluid resuscitation, complications such as pulmonary edema may develop, highlighting the need for continuous clinical monitoring.
- Judicious, goal-directed hydration and awareness of potential fluid overload are key to optimizing outcomes in the management of rhabdomyolysis.

Rhabdomyolysis carries a high risk of acute kidney injury (AKI)^[^5^]^. If left unaddressed, subsequent electrolyte abnormalities and metabolic acidosis can lead to severe complications such as cardiac arrhythmias and compartment syndrome^[^2^]^. Therefore, early recognition and aggressive fluid resuscitation are critical: restoring renal perfusion and diluting intratubular myoglobin markedly reduce the risk of permanent damage. Prompt IV hydration is considered the keystone of therapy to prevent AKI^[^6^]^. Our case presents an unusual etiology of exercise-induced rhabdomyolysis (EIR), emphasizes the need to consider rhabdomyolysis even following atypical exertion, and clarifies that timely diagnosis with aggressive supportive care can yield excellent outcomes.

This work has been reported in line with the CARE^[^7^]^ and the TITAN reporting guidelines^[^8^]^.

## Case presentation

The patient, a 34-year-old male with no prior medical history, presented with generalized myalgia, cola-colored urine, and an episode of vomiting. He reported participating in a swimming session lasting approximately 2 hours in hot weather; he felt well beforehand and became symptomatic about 2 hours after finishing. He denied chronic illnesses, regular medications, or use of herbal remedies, except for a single dose of ibuprofen taken after symptom onset. The patient denied any recent viral illness, including upper respiratory or gastrointestinal infections, prior to the exertional event. No personal or familial history suggestive of metabolic or hereditary myopathies was identified. On arrival, his vital signs were stable: temperature 37.1°C, blood pressure 120/78 mmHg, heart rate 102 beats/min, respiratory rate 20 breaths/min, and SpO_2_ 96% on room air.

Initial laboratory evaluation showed a markedly elevated creatine phosphokinase (CPK), which peaked at 250 000 U/L, elevated transaminases and lactate dehydrogenase (LDH), and a serum creatinine of 2.5 mg/dL. Urinalysis demonstrated 3+ blood on dipstick in the absence of red blood cells on microscopy, consistent with myoglobinuria; urine myoglobin was subsequently confirmed to be markedly elevated at 4800 ng/mL. Serum electrolytes revealed hyperkalemia (potassium 5.8 mEq/L), hyperphosphatemia (phosphorus 6.2 mg/dL), hypocalcemia (calcium 7.4 mg/dL), and an elevated uric acid level of 9.1 mg/dL. Repeat laboratory testing several hours later demonstrated a rise in serum creatinine to 5.0 mg/dL. The patient was started on aggressive volume resuscitation with 3 L of isotonic normal saline. A Foley catheter was placed, and a continuous infusion of isotonic saline was continued and titrated to maintain a urine-output target of approximately 2 mL/kg/hr. The patient also received pantoprazole during initial therapy.

Because of progressive renal impairment and the severity of rhabdomyolysis, he was admitted to the Medical Intensive Care Unit for close monitoring and further management. On the following day, he developed pulmonary edema attributed to fluid overload; this was managed with high-flow nasal cannula oxygen and intravenous furosemide. By intensive care unit (ICU) day 2, his creatinine had risen to 10 mg/dL, and CPK, LDH, and liver enzymes remained markedly elevated, as shown in Table 1. Repeat laboratory testing 7 days after ICU admission showed that serum creatinine peaked at 14 mg/dL. Despite the peak creatinine level, he did not meet standard indications for renal replacement therapy (RRT). Therefore, RRT was not initiated.

**Table 1:** Laboratory values during hospitalization and follow-up.

Variable (units)	Day 1	Day 2	Day 7	Day 9	Follow-up (2 weeks)
Creatine kinase (U/L)	257 321	231 700	62 300	6100	244
Myoglobin-MB (U/L)	49 237	27 824	9541	821	38
Aspartate transaminase (AST) (U/L)	1159	1277	421	133	38
Alanine aminotransferase (ALT) (U/L)	627	681	320	181	42
Lactate dehydrogenase (LDH) (U/L)	1820	1560	720	360	180
Serum urea (mg/dL)	66	95	160	77	13
Serum creatinine (mg/dL)	2.50	10.0	14.0	3.70	1.08

With continued ICU management, including careful fluid balance, diuresis, electrolyte correction, nephrology input, and close urine-output monitoring, the patient gradually improved. By ICU day 9, his creatinine had trended down to 3.7 mg/dL; CPK and liver tests were decreasing; electrolytes had normalized (potassium 4.2 mEq/L, calcium 8.6 mg/dL); and his oxygen saturation was satisfactory on room air. Because he was hemodynamically stable and clinically improved, he was transferred to the medical ward for continued recovery.

Four days later, he was clinically well, and laboratory parameters had improved: CPK had fallen to 711 U/L, and renal function continued to trend downward, so he was discharged home. He was advised on adequate hydration, avoidance of strenuous exercise, and close nephrology follow-up with daily serum creatinine monitoring. At an outpatient review 2 weeks after discharge, his serum creatinine had declined to 1.08 mg/dL.

## Discussion

Rhabdomyolysis is a syndrome of skeletal muscle breakdown resulting in the release of myoglobin, CPK, potassium, and uric acid into the bloodstream. Patients usually present with muscle pain (myalgias), generalized weakness, and dark tea-colored urine from myoglobinuria^[^9^]^. Various etiologies of rhabdomyolysis have been reported. In adults, the most common causes were trauma, drugs, and infections^[^6^]^. Several risk factors predispose to rhabdomyolysis, including male sex, African-American race, very young patients (<10 years), older adults (>60 years), and extreme obesity (body-mass index exceeding 40 kg/m^2^)^[^6^]^. However, our patient was previously healthy with no significant past medical history and denied taking illicit substances or any medications prior to the episode; his only risk factor was being male.

EIR is a well-documented but relatively uncommon phenomenon, typically reported after sudden initiation of intense physical activity, heavy or unaccustomed exercise, or exercise performed for a prolonged duration^[^10,11^]^. In contrast, our patient developed severe rhabdomyolysis following a swimming session, an exertion not usually associated with such profound muscle breakdown. Creatine kinase (CK) levels in ER can rise markedly above the normal upper limit and may reach into the tens of thousands^[^10,11^]^. In our case, the patient’s CK peaked at approximately 250 000 U/L, indicating extensive skeletal muscle injury despite modest physical exertion and the absence of predisposing comorbidities and risk factors. The disproportionate degree of muscle injury likely reflects a confluence of factors: prolonged, unaccustomed exertion; heat-related muscle stress; and possible individual susceptibility, suggesting that factors beyond exercise intensity alone contributed to the severity of muscle injury in this patient.

The severe degree of rhabdomyolysis observed in our patient is rather unexpected given the apparently moderate nature of the exercise. One possible explanation is the combination of prolonged swimming and exposure to hot weather. Although swimming is generally considered a lower-risk activity for ER, environmental heat exposure may increase physiological stress and contribute to dehydration, potentially augmenting exercise-related muscle injury. Individual susceptibility may also have played a role. Therefore, the combination of prolonged exertion, heat exposure, and relative dehydration may explain the unusually severe presentation in this case^[^4,6^]^.

AKI is a well-recognized, serious complication of rhabdomyolysis that occurs in approximately 13–50% of patients, depending on the definitions used and clinical scenarios^[^5^]^. The mechanisms underlying AKI are multifactorial: renal vasoconstriction from intravascular volume depletion, intratubular cast formation, and myoglobin toxicity in the kidneys resulting in acute renal failure^[^6,9^]^. Our patient developed AKI according to KDIGO criteria, with a rise in serum creatinine exceeding 0.3 mg/dL within 48 hours^[^12^]^. The degree of renal dysfunction was profound, as the serum creatinine peaked at 14 mg/dL despite initial supportive measures. Notably, the patient took a single dose of ibuprofen after symptom onset; while a causal role cannot be established from a single case, nonsteroidal anti-inflammatory drugs are known to impair renal perfusion through prostaglandin inhibition and may exacerbate AKI in the setting of rhabdomyolysis^[^13^]^.

Management of rhabdomyolysis centers on aggressive intravenous hydration to restore renal perfusion and increase urine flow, thereby reducing the risk of developing AKI^[^6^]^. Fluid resuscitation should not be “blind” – fluids are best titrated to a urine-output target (commonly cited targets are approximately 1–3 mL/kg/hr or ≈ 200–300 mL/hr) and continued until CK and myoglobinuria are clearly downtrending^[^14^]^. In our patient, a Foley catheter was placed, and fluids were titrated to maintain an approximate target urine output of 2 mL/kg/hr. Clinicians must balance aggressive volume repletion with the risk of iatrogenic fluid overload: pulmonary edema is a recognized complication of liberal IV fluids in patients with rhabdomyolysis, particularly when renal clearance is impaired, and, therefore, close clinical and respiratory monitoring is essential. Loop diuretics and oxygen support (including high-flow nasal cannula) are appropriate measures when volume overload compromises gas exchange^[^15,16^]^.

RRT is indicated for the usual AKI complications (refractory hyperkalemia, severe metabolic acidosis, overt fluid overload not responsive to diuretics, or uremic complications) rather than on the basis of creatinine or myoglobin values alone^[^5,9^]^. Thus, a markedly elevated serum creatinine alone is not an absolute indication for dialysis if the patient maintains urine output and has no refractory electrolyte or acid-base disturbances, a rationale that explains the conservative approach taken in this case.

## Conclusion

Rhabdomyolysis is a potentially life-threatening syndrome with varied etiologies and clinical presentations. ER, while uncommon, can occur even in healthy individuals after relatively modest physical activity. This case highlights three practical points:^[^1^]^ early aggressive fluid therapy remains the cornerstone of management but should be titrated to a urine-output target^[^2^]^, monitoring for and promptly treating fluid overload (including pulmonary edema) is essential during aggressive resuscitation^[^3^]^, and even very severe biochemical abnormalities can sometimes be managed conservatively when standard dialysis indications are absent – but such presentations are rare and warrant multidisciplinary critical care and nephrology input.

## Data Availability

The data supporting the findings of this study are available from the corresponding author upon reasonable request.
